# Smart Hydrogen Atoms in Heterocyclic Cations of 1,2,4-Triazolium-Type Poly(ionic liquid)s

**DOI:** 10.1021/acs.accounts.2c00430

**Published:** 2022-12-05

**Authors:** Si-hua Liu, Hong Wang, Jian-ke Sun, Markus Antonietti, Jiayin Yuan

**Affiliations:** † MOE Key Laboratory of Cluster Science, Beijing Key Laboratory of Photoelectronic/Electrophotonic Conversion Materials, School of Chemistry and Chemical Engineering, Beijing Institute of Technology, Beijing 102488, P. R. China; ‡ Department of Colloid Chemistry, Max-Planck Institute of Colloids and Interfaces, Research Campus Golm, Am Mühlenberg 1, 14476 Potsdam, Germany; § Key Laboratory of Functional Polymer Materials (Ministry of Education), Institute of Polymer Chemistry, College of Chemistry, Nankai University, Tianjin 300071, P. R. China; ∥ Department of Materials and Environmental Chemistry, Stockholm University, Stockholm 10691, Sweden

## Abstract

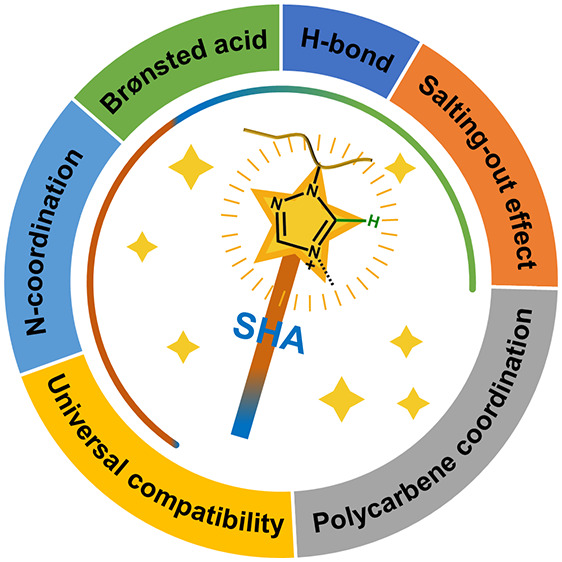

Discovering and constructing molecular functionality platforms for materials chemistry innovation has been a persistent target in the fields of chemistry, materials, and engineering. Around this task, basic scientific questions can be asked, novel functional materials can be synthesized, and efficient system functionality can be established. Poly(ionic liquid)s (PILs) have attracted growing interest far beyond polymer science and are now considered an interdisciplinary crossing point between multiple research areas due to their designable chemical structure, intriguing physicochemical properties, and broad and diverse applications. Recently, we discovered that 1,2,4-triazolium-type PILs show enhanced performance profiles, which are due to stronger and more abundant supramolecular interactions ranging from hydrogen bonding to metal coordination, when compared with structurally similar imidazolium counterparts. This phenomenon in our view can be related to the smart hydrogen atoms (SHAs), that is, any proton that binds to the carbon in the N-heterocyclic cations of 1,2,4-triazolium-type PILs. The replacement of one carbon by an electron-withdrawing nitrogen atom in the broadly studied heterocyclic imidazolium ring will further polarize the C–H bond (especially for C5–H) of the resultant 1,2,4-triazolium cation and establish new chemical tools for materials design. For instance, the H-bond-donating strength of the SHA, as well as its Bro̷nsted acidity, is increased. Furthermore, polycarbene complexes can be readily formed even in the presence of weak or medium bases, which is by contrast rather challenging for imidazolium-type PILs. The combination of SHAs with the intrinsic features of heterocyclic cation-functionalized PILs (e.g., N-coordination capability and polymeric multibinding effects) enables new phenomena and therefore innovative materials applications.

In this Account, recent progress on SHAs is presented. SHA-related applications in several research branches are highlighted together with the corresponding materials design at size scales ranging from nano- to micro- and macroscopic levels. At a nanoscopic level, it is possible to manipulate the interior and outer shapes and surface properties of PIL nanocolloids by adjusting the hydrogen bonds (H-bonds) between SHAs and water. Owing to the interplay of polycarbene structure, N-coordination, and the polymer multidentate binding of 1,2,4-triazolium-type PILs, metal clusters with controllable size at sub-nanometer scale were successfully synthesized and stabilized, which exhibited record-high catalytic performance in H_2_ generation via methanolysis of ammonia borane. At the microscopic level, SHAs are found to efficiently catalyze single crystal formation of structurally complex organics. Free protons *in situ* released from the SHAs serve as organocatalysts to activate formation of C–N bonds at room temperature in a series of imine-linked crystalline porous organics, such as organic cages, macrocycles and covalent organic frameworks; meanwhile the concurrent “salting-out” effect of PILs as polymers in solution accelerated the crystallization rate of product molecules by at least 1 order of magnitude. At the macroscopic scale, by finely regulating the supramolecular interactions of SHAs, a series of functional supramolecular porous polyelectrolyte membranes (SPPMs) with switchable pores and gradient cross-sectional structures were manufactured. These membranes demonstrate impressive figures of merit, ranging from chiral separation and proton recognition to switchable optical properties and real-time chemical reaction monitoring. Although the concept of SHAs is in the incipient stage of development, our successful examples of applications portend bright prospects for materials chemistry innovation.

## Key References

- SunJ.-K.; KochovskiZ.; ZhangW.-Y.; KirmseH.; LuY.; AntoniettiM.; YuanJ.General synthetic route toward highly dispersed metal clusters enabled by poly(ionic liquid)s. J. Am. Chem. Soc.2017, 139 ( (26), ), 8971–89762858583510.1021/jacs.7b03357.^1^*In situ generated polycarbenes of 1,2,4-triazolium-type poly(ionic liquid)s under mild base conditions enabled strict size control (approximately 1 nm) over a broad range of metal clusters, which showed record-high catalytic performance.*
- SunJ.-K.; ZhangW.; GutermanR.; LinH.-J.; YuanJ.Porous polycarbene-bearing membrane actuator for ultrasensitive weak-acid detection and real-time chemical reaction monitoring. Nat. Commun.2018, 9, 17172971289910.1038/s41467-018-03938-xPMC5928224.^2^*Polycarbene-laden 1,2,4-triazolium-type poly(ionic liquid) porous membrane actuators featuring two concurrent structural gradients of the electrostatic complexation degree and the carbene–NH_3_ adduct density along the membrane cross-section were constructed and display the highest sensitivity to aqueous media protons (10^–6^ mol L^–1^) and the ability to monitor the entire chemical reaction process.*
- ShaoY.; WangY. L.; LiX.; KheirabadA. K.; ZhaoQ.; YuanJ.; WangH.Crosslinking of a single poly(ionic liquid) by water into porous supramolecular membranes. Angew. Chem., Int. Ed.2020, 59, 17187–1719110.1002/anie.20200267932583932.^3^*Through hydrogen-bond-induced phase separation of a single-component poly(ionic liquid) between its polar and apolar domains, a general one-step method to supramolecular porous polyelectrolyte membranes with switchable porosity from a single-component poly(ionic liquid) was established.*
- ZhangS.-Y.; MiaoH.; ZhangH.-M.; ZhouJ.-H.; ZhuangQ.; ZengY.-J.; GaoZ.; YuanJ.; SunJ.-K.Accelerating crystallization of open organic materials by poly(ionic liquid)s. Angew. Chem., Int. Ed.2020, 59, 22109–2211610.1002/anie.202008415PMC775645832748542.^4^*1,2,4-Triazolium-type poly(ionic liquid)s could serve as a universal additive to accelerate by at least 1 order of magnitude the growth rate of representative imine-linked crystalline porous organics, including organic cages, covalent organic frameworks, and macrocycles, resulting from the active C5-proton in poly(1,2,4-triazolium) that catalyzes the formation of imine bonds and the simultaneous salting-out effect.*

## 1 Introduction

Poly(ionic liquid)s (PILs) are the polymerization products of ionic liquids (ILs). They have attracted growing interest as they can serve as an interdisciplinary topic among multiple research areas (e.g., chemical synthesis, materials science, and nanotechnology) due to high freedom in chemical structure design through the utilization of abundant IL monomer units and the related intriguing physicochemical properties.^5−8^ PILs carrying 1,3-dialkylimidazolium cations are the prototypical and so far most studied N-heterocyclic polymers and display broad and diverse uses.^9−11^ Surprisingly, when imidazolium- and 1,2,4-triazolium-type PILs are compared, the isoelectronic substitution of the methylidyne group by nitrogen (i.e., aza substitution) led to enhanced polarization of the C–H bonds on the heterocyclic cations, which strengthens the supramolecular interactions of PILs in terms of H-bond formation and polycarbene complexation.^1−4,12^ Poly(1,2,4-triazolium)s are composed of polymeric backbones bearing a 1,2,4-triazolium repeating unit and counteranions and offer options for unexpected supramolecular interactions and organocatalysis, such as H-bond-induced self-assembly^12^ or phase separation,^3^ polycarbene–metal coordination,^1^ and catalysis by N-heterocyclic carbenes.^1,4^ Our recent systematic studies have revealed that most of these diverse functions are derived from the eminently polarized C–H bonds in these cations. The active hydrogen atom in the 1,2,4-triazolium cation might constitute an ideal site for chemical assembly tools for materials engineering. Herein, we attempt to share our view on the roles of smart hydrogen atoms (SHAs) in shaping the property profile and enabling a myriad of applications of 1,2,4-triazolium-PILs. The SHAs here are specifically defined as the active hydrogen atoms in the N-heterocyclic cations and are bonded to the highly polarized carbon atom (C3/C5) between two nitrogens (i.e., N=***C***(H)–N). In this Account, we focus exclusively on the SHA tethered to the C5 carbon, because its SHA effect is more prominent.

The topic of SHAs in heterocyclic cations is based on our latest advancements in their synthesis and materials applications, highlighting the unusual physicochemical properties and supramolecular interactions, which could set the first cornerstone and reference guide for SHA chemistry yet to come. The discussion starts with the fundamentals coming with the chemical structure using 1,2,4-triazolium PILs as a prototypical case. Next, applications are showcased and interspersed by a look into our recent individual work, including polymeric nanoparticle self-assembly and metal cluster stabilization, acceleration of crystallization, and porous PIL membrane fabrication. Finally, the current challenges and future trends in investigating and utilizing SHAs in N-heterocyclic PILs and beyond are proposed.

## 2 Structure and Properties of SHAs

**Figure 1:**
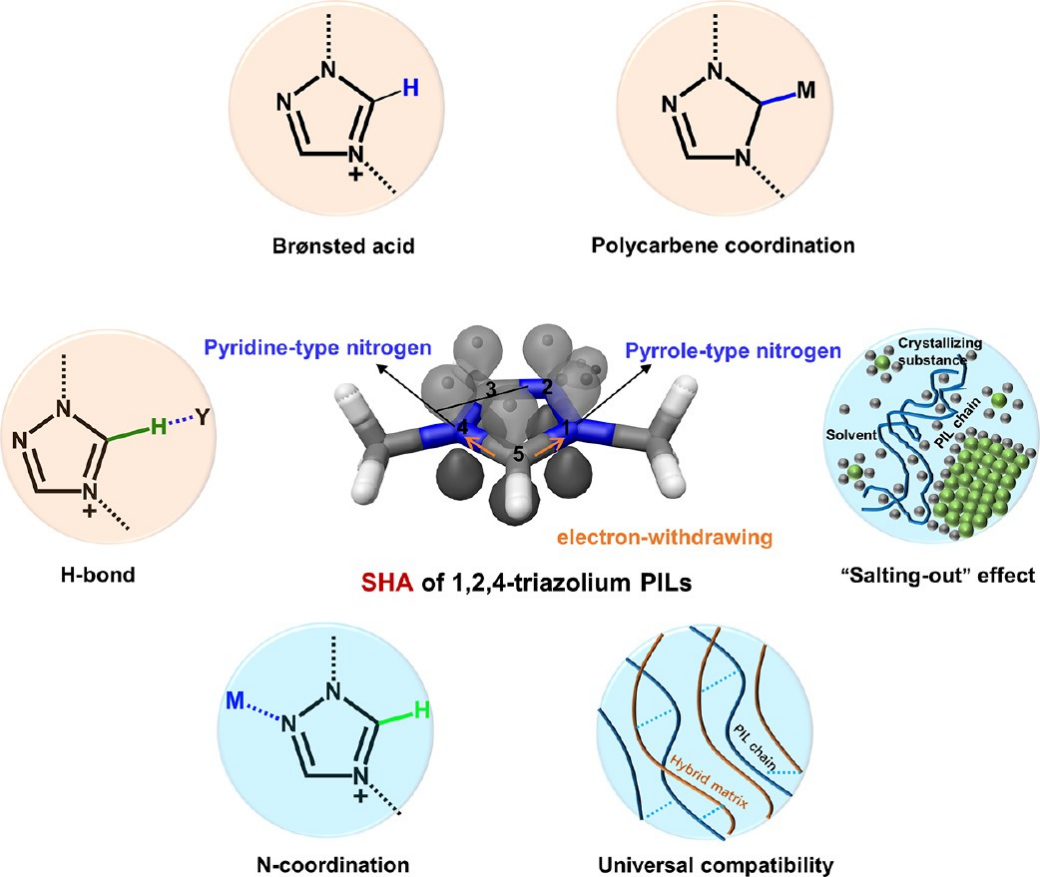
Electronic structure and the features of the N-heterocyclic cation in the 1,2,4-triazolium-ring. Typical supramolecular interactions of SHAs and properties of N-heterocyclic PILs are highlighted by pink and light blue discs, respectively.

This section is dedicated to structure-based fundamentals and physicochemical properties of N-heterocyclic cations in PILs (Figure 1). For simplicity, we continuously compare the less studied 1,2,4-triazolium cation and the well-known imidazolium cation to rationalize the intriguing profile of SHAs from the viewpoint of fundamental electronic effects.

### 2.1 Electronic Structure of the N-Heterocyclic Cations

According to the basic theory of heterocyclic chemistry and as judged by electron density and polarization, the five-membered imidazole ring possesses one pyridine-like nitrogen atom and one pyrrole-type nitrogen atom, while the triazole ring features two pyridine-type nitrogen atoms and one pyrrole-type nitrogen atom.^13^ All the ring atoms (i.e., carbon and nitrogen atoms) follow the sp^2^ hybridization model and jointly define the cation’s chemical profile. The pyridine-type nitrogen (−N=, N2 and N4) features a σ lone pair in an sp^2^ hybridization orbital, which protrudes from the ring perpendicular to the π-bond system; such a pyridine-type nitrogen is strongly π-accepting and weakly σ-donating in comparison to the other ring atoms.^14^ The pyrrole-type nitrogen offers a lone pair that is a part of the aromatic π sextet. Thus, the pyrrole-type nitrogen (N1) is π-donating and σ-accepting, leading to an aromatic system of π-electrons.^14^ Considering the higher electronegativity of N than C, polarization of the aromatic π system and the σ framework increases upon successive aza substitution.^14,15^ Undoubtedly, the polarization of the C–H (C5) tethered to the heterocyclic ring is the strongest in the position adjacent to the σ-accepting, pyrrole-type nitrogen.^16^ Upon the introduction of an electron-withdrawing nitrogen atom into the imidazolium ring, the C–H in the 1,2,4-triazolium cation becomes more polarized. Logically speaking, the C–H in the 1,2,4-triazolium ring is expected to undergo a variety of intriguing chemical interactions that differ from those in the imidazolium ring; the latter has been so far intensively investigated due to commercial availability of imidazolium-type ionic liquids, leaving the former rather unexplored.

### 2.2 Brønsted Acid–Base Behavior and Hydrogen Bonds of SHAs

The Brønsted acid–base properties of the N-heterocyclic cations allow us to describe the supramolecular behavior of PIL-based functional materials. When we compare benzene, pyridine, pyrrole, imidazole, and triazole in a series, successive aza substitution increases the ionic character in the C–H covalent bond, that is, it becomes more prone to releasing the H as a proton.^16−18^ For example, according to a density functional theory (DFT) investigation, the p*K*_a_ values in DMSO for C–H deprotonation of 1-methyl-1,2,4-triazole are 34.0 (C3) and 28.5 (C5), while those of 1-methyl-imidazole are 34.1 (C2), 39.8 (C4), and 34.2 (C5).^16^ Consistent with the principle of polarization, the C–H bond adjoining the σ-accepting, pyrrole-type nitrogen possesses the highest acidity. The p*K*_a_ of the corresponding azoliums with quaternized N follows a similar trend. The presence of the additional electron-withdrawing nitrogen atom in the cationic ring will make the system even more electron deficient and destabilize the 1,2,4-triazolium ion relative to its formally neutral transition state, thereby increasing the acidity by some orders of magnitude.^14,19^ Studies of the proton transfer reactions of a range of triazolyl carbenes indeed indicate that 1,2,4-triazolium is more acidic by 5 p*K*_a_ units than analogous imidazolium architectures.^20^

Hydrogen bonds, with bond strengths typically ranging from 4 to 40 kJ mol^–1^, are among the most important inter-/intramolecular interaction patterns governing self-assembly from microscopic to macroscopic materials.^21^ According to the definition by the International Union of Pure and Applied Chemistry (IUPAC), a hydrogen bond, R–H···Y, is an attractive interaction in which the hydrogen atom is positively polarized by an electronegative species, R (element or group), and linked to Y (atom, ion, or molecule) via electron-rich regions, mostly lone pairs.^22−24^ Accordingly, the polarized C–H bonds in the N-heterocyclic cations are H-bond donors, especially valid for the C5–H in the 1,2,4-triazolium rings. It is noteworthy that the extrinsically polarized C–H bond can provide hydrogen bonds that are as strong as those of classical intrinsically polarized hydrogen bond donors (i.e., N–H and O–H). Importantly, the relative character of H-bonds established by the C–H bonds is different considering the covalent and ionic contributions. The ionic contribution to the H-bond parallels the acidity of the H-bond donor as polarization of the R–H bond is required for H-bond donation. As the C5–H bond in the 1,2,4-triazolium is more polarized than any C–H bond in the 1,3-dialkylimidazolium, the energy of the σ*(C5–H) orbital is lower, and more electron density is shifted to the carbon to activate the hydrogen. This enables a stronger hyperconjugative n(Y) → σ*(C5–H) charge-transfer interaction, that is, C5–H···Y.^25−27^ Thus, the strength of H-bonds donated by C–H bonds of 1,2,4-triazolium rings is higher than that of H-bonds donated by 1,3-dialkylimidazolium rings, and furthermore, the 1,2,4-triazolium ring still carries an untouched pyridinic nitrogen, being simultaneously a H-bridge acceptor.

### 2.3 N-Heterocyclic Polycarbene Precursors

N-Heterocyclic carbenes (NHCs) have ranked among the most vigorous tools in coordination chemistry and organocatalysis since the first isolation of stable NHCs in 1991.^28−31^ The lone pair σ-electrons located in the plane of the heterocyclic ring endow these NHC compounds with superior nucleophilicity. Consequently, the NHCs as σ-donors can bind to a broad spectrum of metallic and nonmetallic species. In an approximation, the trend of carbene donor strength of the corresponding heterocyclic cations is dominated by the number of iminium-type nitrogen atoms and their vicinity to the C–H group.^14^ The main approaches for preparation of the classic imidazolium-based NHCs require an inert atmosphere and anhydrous environment with a strong base, such as sodium hydride or specifically synthesized precursors (e.g., CO_2_ adducts).^32−35^ Such sophisticated reagents and the coupled protection measures restrict the applications of NHCs. Excitingly, deprotonation of 1,2,4-triazolium precursors occurs under significantly more convenient conditions. This is due to the introduction of the additional electron-withdrawing nitrogen atom, which destabilizes the parent 1,2,4-triazolium cation to make the deprotonation thermodynamically more favored. As a typical example, the C5–H bond in poly(1,2,4-triazolium)s could be easily deprotonated and subsequently form active polycarbene complexes already under weak-to-medium base conditions in an aqueous phase.^2^ Another attractive feature of N-heterocyclic polycarbenes is the combined merits of carbene and polymeric materials, which open up a much unexplored area of carbene chemistry for materials applications (*vide infra*).

### 2.4 Cooperativity of Binding with SHAs

The nitrogen atoms with lone pairs present in the 1,2,4-triazolium cations (but absent in the imidazolium cation) as coordination sites can bind metal species via coordination bonds through empty d or f orbitals of transition metals.^36^ The polymeric chain with diverse active species originating from SHAs and conventional coordination sites might establish a constructive cooperativity of supramolecular interactions, for example, polycarbene coordination coexists and is supported by N coordination to better stabilize objects with multiple binding motifs, such as metal surfaces or metal clusters.^1^ In addition, the polymeric nature of N-heterocyclic PILs and their liquid character in a wide temperature range makes them readily processable, similar to common thermoplasts, for practical use. The solubility of PILs in aqueous or oily phases can be flexibly adjusted by the chosen counteranions that are exchangeable via ion metathesis.

Due to better interactions of various types, PIL chains first bind to themselves but also bind strongly to solvent molecules (“solvation”) and change solvent structure. They compete with other substances for solvent molecules and thereby decrease their solubility and even lead to their “phase-out”. The resulting precipitates could subsequently be stabilized as colloidal microphases by the same PIL chains. Recently, such merit was employed for the rapid growth of large and high-quality crystals under a shear flow.^37^

Due to the structure formation involved in PILs, 1,2,4-triazolium PILs are highly complex in internal structures and comparably low in entropy, and interaction with other self-structured molecules, such as water, can improve or lower that. Thermodynamics is mostly not enthalpy- but entropy-driven, and for water, this is known as the “hydrophobic effect”, with the Hofmeister effect and the Hofmeister series being the potentially best known consequence.

Due to their abundant ionic interaction sites, high polarizability, hydrophobic molecular subunits, plus strong self-organization properties and the coupled “hydrophobic effect”, PILs can interact with a wide range of species, including water, hydrophobic oils, organics, inorganics, biomaterials, and their hybrids. As such, 1,2,4-triazolium PILs enable functional materials design, which is why PILs even with less SHA character, that is, polyimidazoliums, have been already described as rather universal dispersants and reaction media.^38^

## 3 SHA Application: From Nano- to Micro- to Macroscopic Scale

### 3.1 SHA-Guided Internal Morphology Control of Polymeric Nanoparticles

Next, interesting application examples based on SHA chemistry are highlighted. The highly polarized C–H bonds in the N-heterocyclic 1,2,4-triazolium cations are strong H-bridge donors, while the N2-nitrogen atom acts as a H-acceptor; this chemical pattern imposes a strong interaction with water, which explains the change in the solvent structure. These characteristics affect the surface energy of water but also its interface energy with different materials. Colloidal particles involving PILs will adapt their structure and self-assembly behavior to meet the new energy balance, allowing them to vary their inner and outer morphologies upon chemical modification of the PILs.

**Figure 2:**
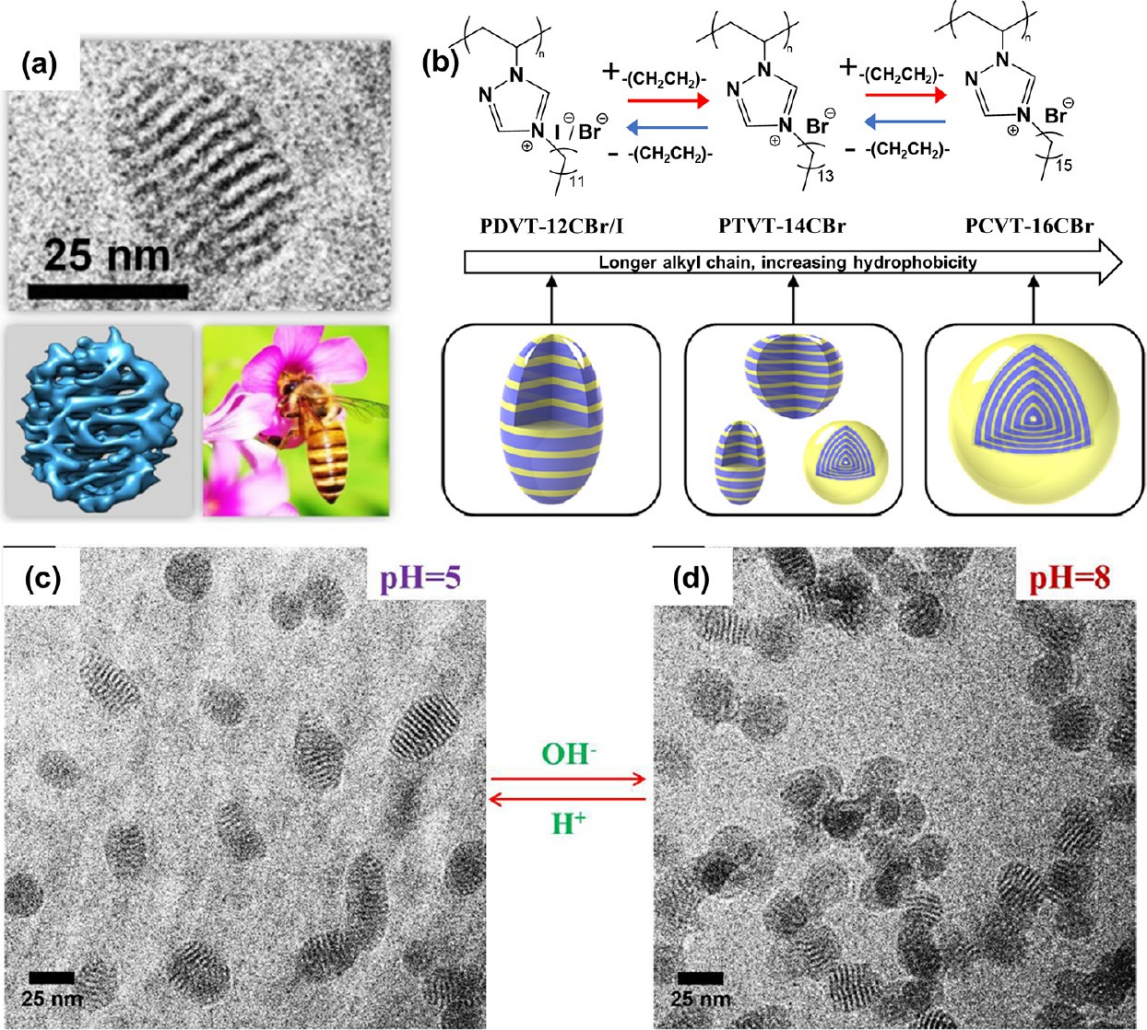
Internal morphology control of PIL nanoparticles with SHAs. (a) Nanoparticle of 1,2,4-triazolium-type PILs with a wasp-like internal structure pattern. (b) Morphological modulation from a wasp-like to an onion-like morphology by increasing the alkyl chain lengths. (c, d) Cryo-electron microscopy images of PIL nanocolloids in aqueous phase with different pH values. Reproduced with permission from ref (12). Copyright 2016 American Chemical Society.

Nanocolloids with an ordered onion-like inner structure were prepared by radical polymerization of 3-dodecyl-1-vinylimidazolium bromide with a long alkyl substituent (termed DVIm-12CBr, chemical structures in Figure S1) in an aqueous phase as the reference system.^39^ When a structurally similar SHA monomer, 4-dodecyl-1-vinyl-1,2,4-triazolium bromide (termed DVT-12CBr, Figure S1) was used under the same polymerization conditions, a morphological variation of nanoparticles from an onion-like (for DVIm-12CBr) to a wasp-like morphology (for DVT-12CBr) was found (Figure 2a).^12^ As the two monomers, DVIm-12CBr and DVT-12CBr, are chemically alike, this morphological deformation means that an apparently “subtle perturbation” in the chemical structure, here specifically by substituting one carbon atom in DVIm-12CBr with a nitrogen atom in DVT-12CBr, induces notable internal structure evolution in their self-assembled nanocolloids. Namely, the SHA site makes the 1,2,4-triazolium unit more hydrophilic. By increase of the alkyl chain length of the 1,2,4-triazolium unit, a structural transition from wasp-like to onion-like morphology was achieved (Figure 2b), which is impossible for the imidazolium counterpart. Moreover, the dispersion behavior of the PDVT-12CBr nanocolloids can be regulated via the solution pH (Figure 2c). When a final pH of 8 was reached, the PDVT-12CBr nanocolloids started to aggregate with each other due to their decreased surface charge density and thus electrostatic colloidal stability. The well-dispersed state can be readily reestablished upon addition of hydrochloric acid solution to drop the pH value to 5. This oscillation between individual and aggregation states illustrates the environment-responsive capability of the PIL nanocolloids (e.g., pH, hydrophobic compounds, but also response according to other interactions), which could be further upgraded into a more functional smart colloidal system via surface grafting.^40^

### 3.2 Stabilization of Metal Clusters through Polycarbene Ligation

Sub-nanometer sized metal clusters have drawn much attention for their extraordinary physicochemical properties and reactivities and various corresponding applications in chemical sensing, fluorescence detection, catalysis, and more.^41−44^ The SHA-PILs enable a strategy that can prepare a range of metal clusters with sub-nanometer size that are desirable but otherwise challenging. NHCs are among the strongest ligands for metal nanoparticle stabilization,^45^ but classic carbene ligands of the imidazolium-type require harsh conditions for carbene formation. 1,2,4-Triazolium-type PILs with SHA sites *in situ* form polycarbene under mild deprotonation conditions, and the polymer character of PILs enables cooperative multidentate binding. This makes 1,2,4-triazolium PILs a unique precursor for easy synthesis of polycarbene–metal complexes.

**Figure 3:**
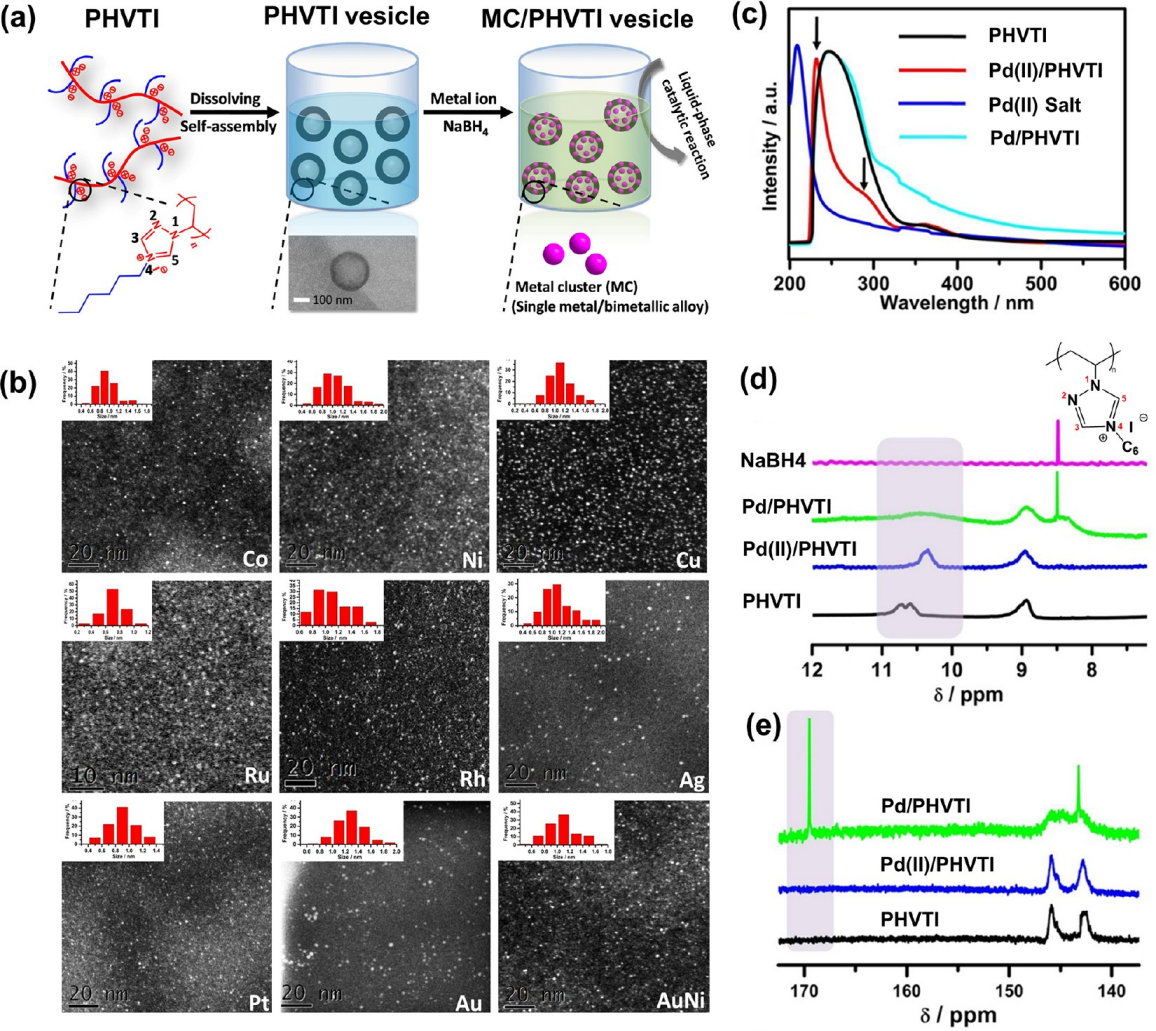
(a) Schematic illustration of the synthesis process of metal clusters with poly(1,2,4-triazolium) vesicles. (b) High-angle annular dark-field scanning transmission electron microscopy images of a series of metal cluster–PHVTI composites. Insets are the size statistical charts of the corresponding metal clusters. (c) UV–vis spectra analyzing the interaction between metal ion (Pd^2+^) and PHVTI in solution. The shift of the absorption band for the metal ion/PHVTI mixture indicated a strong affinity between the metal ion and PIL. (d) ^1^H NMR spectra and (e) ^13^C NMR *in situ* monitoring of the synthesis process of Pd clusters in the polycarbene-bearing vesicle system (CD_2_Cl_2_/CH_3_OH = 2:1). The signal of the C5-proton in the 1,2,4-triazolium cation is at 10.6 ppm. The typical shift of metal–carbene coordination is at around 169.0 ppm. Reproduced with permission from ref (1). Copyright 2017 American Chemical Society.

As an example, PIL poly(4-hexyl-1-vinyl-1,2,4-triazolium iodide) (termed PHVTI, Figure S1) nanovesicles were prepared and exerted strict size control over a series of metal clusters approximately 1 nm in size (Figure 3), including transition metals (e.g., Co, Ni, Cu, Ru, Rh, Ag, Pt, and Au) and even their alloys (e.g., Au/Ni).^1^ According to the scanning transmission electron microscopy (STEM) analysis, the metal clusters are well-dispersed (Figure 3b). The PHVTI-stabilized metal cluster composites prominently exposed and supported the catalytic performance of metal clusters, as exemplified by a model reaction of methanolysis of ammonia borane.^1^

The role of PHVTI in the stabilization and immobilization of metal clusters to polymer nanovesicles was systematically investigated. A strong affinity between metal ions and PHVTI was found, especially the coordinative interplay with the C5-proton was highlighted. A shift of the absorption band was observed for the metal ion/PHVTI mixture compared to either of them, indicative of a strong affinity between the metal ion and the PIL vesicular support in solution (Figure 3c). Integration of the ^1^H NMR spectra indicated that 33% of the 1,2,4-triazolium units in PHVTI participated in metal–carbene formation after addition of NaBH_4_. The formation of the metal–carbene composite was further evidenced by ^13^C NMR spectra (Figure 3e).^1^ Control experiments demonstrated that the employment of imidazolium PIL poly(3-hexyl-1-vinyl-imidazolium iodide) (termed PHVImI, Figure S1) for the same reaction only resulted in metal nanoparticles with larger diameters in a broad range from 1.5 to 11 nm. These data manifested that the *in situ* formed polycarbene is of prime importance for accurate size control and stabilization of the ultrasmall metal clusters; by contrast the C-proton (i.e., N=C(H)–N) of the imidazolium cation is practically inactive for metal–carbene formation under the same conditions as those for the 1,2,4-triazolium. The latter is rather universally compatible with other materials and solution-processable, and it can be composited with substrates in various morphologies. Thus, the synthesis of metal clusters could be promisingly extended from nanovesicles to a range of surfaces and even porous skeletons, enabling surface/interface engineering of the metal cluster active layer for catalysis, sensing, and antimicrobial applications.

### 3.3 SHAs Catalyzing Synthesis and Controlling Crystallization

SHAs can interact with lone pairs or other proton acceptors through strong H-bonds; meanwhile, they can deprotonate readily. In a synergistic reaction model, the reactants would be enriched via their H-bond interaction with the SHAs of the PIL chain, and next the *in situ* formed free protons could catalyze a possible reaction by temporal transfer.

**Figure 4:**
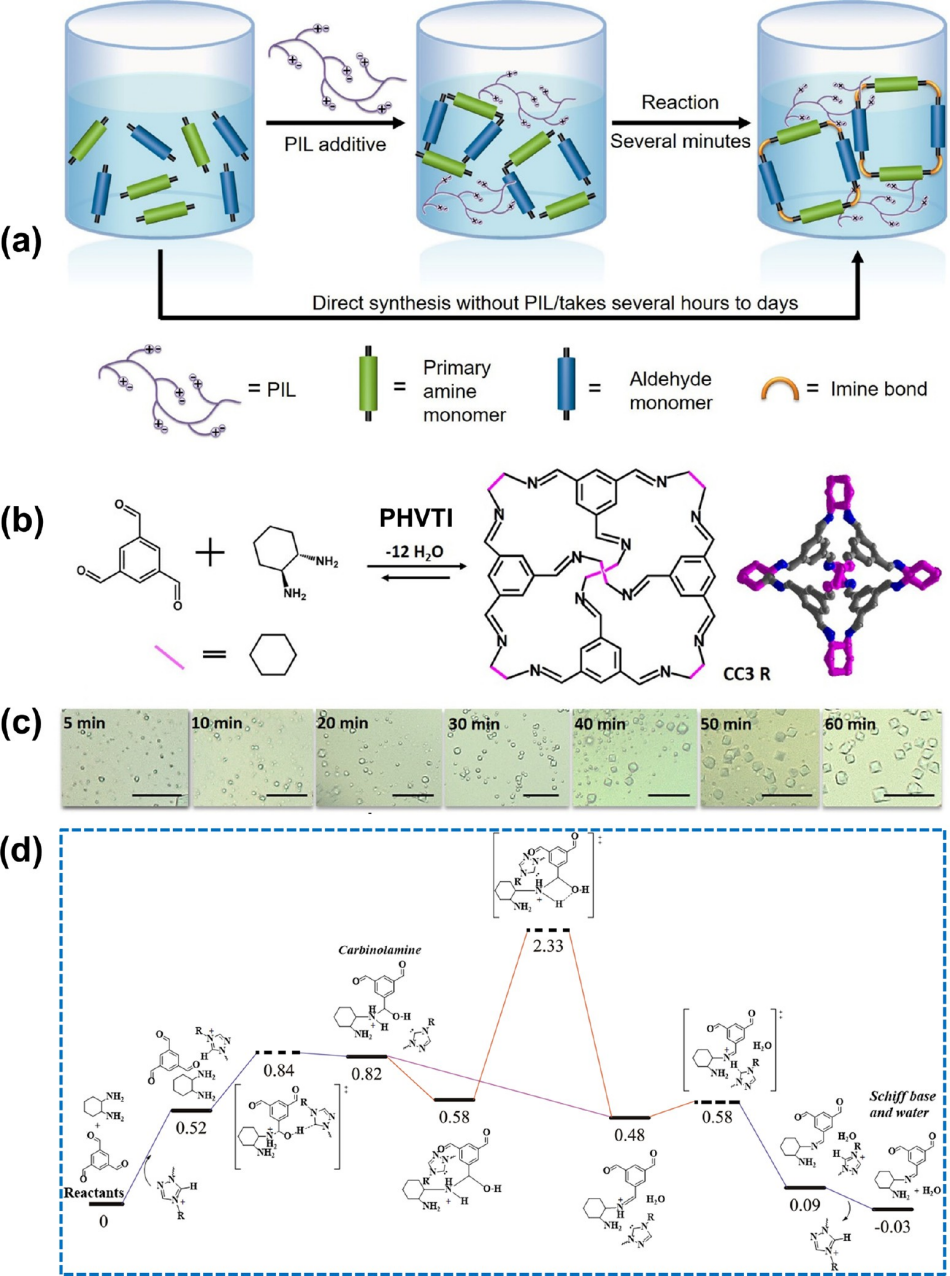
(a) Diagram of accelerating the crystal synthesis of imine bond-linked porous organic materials by PILs. (b) Synthetic process for porous organic cage CC3R. (c) Digital images of the CC3R single crystals with reaction time accelerated by PHVTI; scale bar, 25 μm. (d) Energy profiles of the reaction path catalyzed by 1,2,4-triazolium. Reproduced with permission from ref (4). Copyright 2020 Wiley-VCH.

Imine-linked crystalline porous materials, including porous organic cages, macrocycles, and covalent organic frameworks (COFs), have been broadly investigated for their promising applications in gas and energy storage, catalysis, and molecular separation.^46−48^ Synthesis of these materials is proton-catalyzed and commonly involves a thermal treatment and a long reaction time (up to days). By using 1,2,4-triazolium-type PILs (PHVTI) as macromolecular catalyst, the coupled condensation/crystallization process was significantly shortened from days to minutes at room temperature, while maintaining high structural order of the resulting crystals (Figure 4a–c).^4^ A topographic synergy is responsible for this acceleration. The monomers of aldehyde and amine are gathered near the PIL chains via supramolecular interactions, and the SHAs catalyze the formation of imine bonds; simultaneously, the PIL chains compete for solvent with the products and thus phase out the products in the form of crystals.^37^ Finally, the PIL chains colloidally stabilize the crystals to direct their growth into high-quality large crystals rather than an aggregated precipitate. The evidence for SHAs catalyzing the crystallization was monitored via time-dependent ^1^H NMR spectra.^4^ The reaction pathway calculation revealed that the nucleophilic amine group first attacks the aldehyde group to produce an unstable carbinolamine intermediate. This step is endothermic (Δ*E* = 0.30 eV) with an energy barrier (*E*_a_) of 0.32 eV. Note that the extended H-bridged solvent networks were not included in the calculation. Further dehydration of the carbinolamine forms a stable imine species, which is proton-catalyzed (the purple line in Figure 4d). This process is energy-barrierless and exothermic (Δ*E* = −0.34 eV), suggesting that the rate-limiting step of the overall reaction is the formation of the C–N bond.

The same synthesis procedures were carried out using imidazolium-type PIL PHVImI and pyridinium-type PIL poly(4-hexyl-1-vinyl-pyridinium iodide) (termed PHVPyI, Figure S1). The crystallization rate follows the order of HVTI (5 min) > PHVImI (30 min) > PHVPyI (8 h), where the time refers to how long it takes for the organic cage crystals to reach an average size of 2 μm. It is reasonable that there is no SHA in the pyridinium ring for catalyzing the imine bond formation, while the C5-proton of 1,2,4-triazolium is far more active than the imidazolium C2-proton. Overall, Brønsted acid of the 1,2,4-triazolium-type PIL is a mild and efficient *in situ* proton catalyst and can be potentially also used to regulate other proton-involving reactions.

### 3.4 SHA-Mediated Construction and Functionalization of Smart Membranes

Owing to their polymeric processability and materials compatibility, PILs or their composites can be processed into various functional materials. Predictably, SHAs will play important roles in the construction and functionalization of the resulting PIL microstructures, for example, controlling the porous membrane formation processes, constructing multigradient structures, and allowing for bionic intelligent design.

#### 3.4.1 PIL Membrane with Switchable Porous Structure via SHA-Induced Phase Separation

Noncovalent interactions (e.g., H-bonding, π–π, and van der Waals interactions) are generally employed to engineer supramolecular smart materials with reversible structures.^49^ Abundant noncovalent supramolecular interactions originating from SHAs empower PILs as a structure tool for the formation and exploration of dynamic porous membranes.

**Figure 5:**
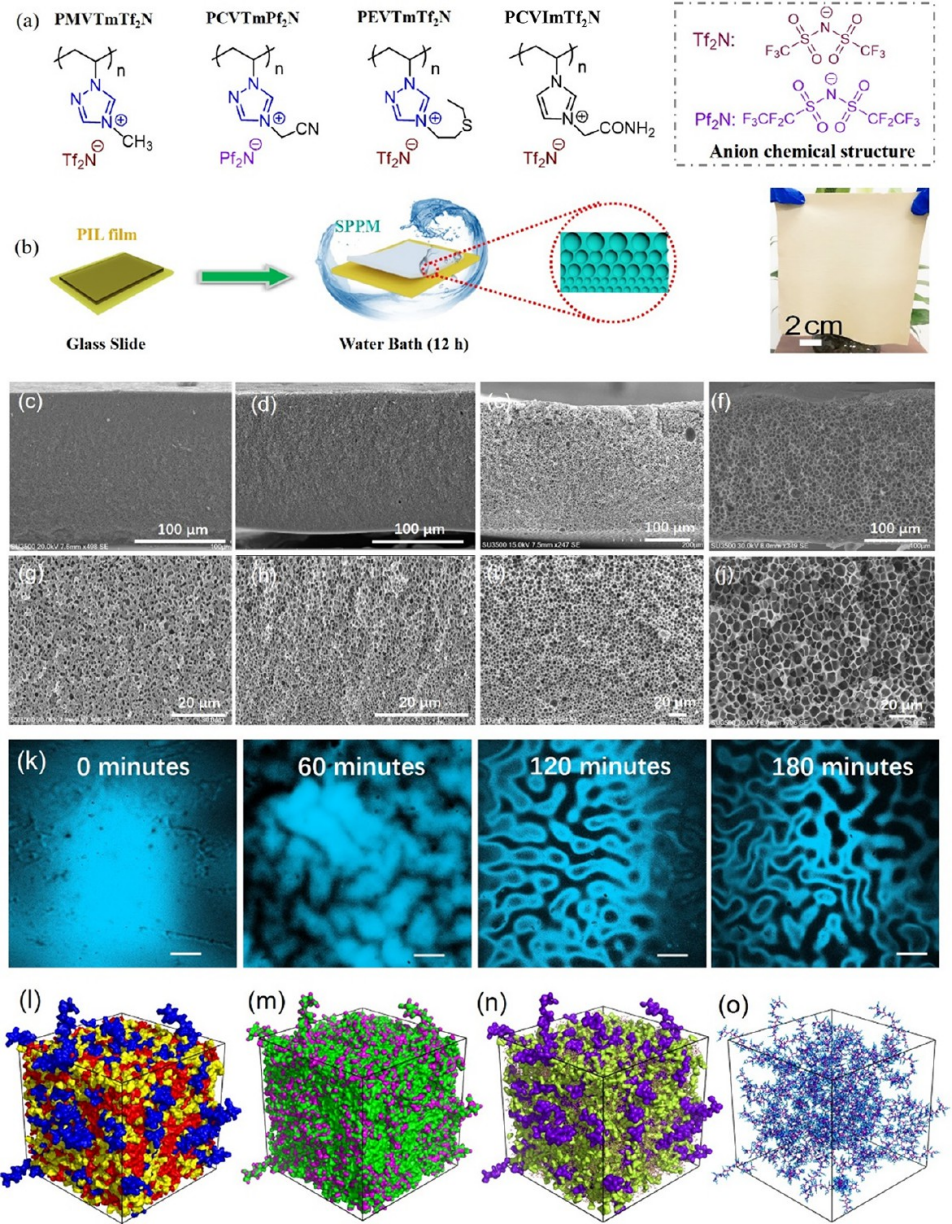
(a) Chemical structures of PILs used to prepare the water-cross-linked SPPMs. (b) Preparation procedure for the SPPMs. The digital image on the right displays a large SPPM with a size of 14 cm × 16 cm. (c–f) Low and (g–j) high magnification cross-sectional scanning electron microscopy images of SPPMs prepared from PMVTmTf_2_N, PCVTmPf_2_N, PEVTmTf_2_N, and PCVImTf_2_N, respectively. (k) Time-dependent laser scanning confocal microscopy image monitoring the water induced microphase separation process. Scale bar, 200 mm. (l–o) Liquid morphologies in SPPMs containing 15% water molecules. Reproduced with permission from ref (3). Copyright 2020 Wiley-VCH.

Recently, we proved that water molecules readily diffuse into the PIL matrix, wherein PIL cations prefer to aggregate via H-bonds and water bridges to form continuous polar domains, while the polymer backbones and the hydrophobic counterions, for example, bis(trifluoromethane sulfonyl)imide (Tf_2_N) and bis(pentafluoroethyl sulfonyl)imide (Pf_2_N), are simultaneously excluded from hydrophilic SHA domains, resulting in microphase separation between the polar and apolar domains in the PIL matrix.^3^ The uptake of water molecules into the membrane gradually decreases due to the increasing mass transfer resistance for water, that is, the film swelling is restricted by the polymer backbones located in the hydrophobic microphase. Finally, a gradient porous architecture of the as-prepared supramolecular porous polyelectrolyte membranes (SPPMs) with decreasing pore size from the top to bottom side is formed (Figure 5). Literally speaking, this work can be considered a breakthrough on the fabrication of SPPMs from a single-component PIL cross-linked by H-bonds with water molecules rather than by complexation and or ionic linkages.

The mechanism of H-bond-induced microphase separation of a PIL network between its polar and apolar domains was confirmed by a range of experimental and theoretical simulation analyses. We found that both the chemical structure of the heterocyclic cation and the alkyl substituent seem to impact the microphase separation behavior and thus the pore development. All three studied 1,2,4-triazolium-type PILs with varied alkyl substituents (PMVTmTf_2_N, PCVTmPf_2_N, and PEVTmTf_2_N, Figure 5a,b) are capable of forming gradient porous structures through the water cross-linking process (Figure 5c–j). However, no freestanding SPPM formed when the poly(vinylimidazolium) counterparts of similar chemical structure (except the replacement of one carbon by one nitrogen atom in the heterocyclic ring) were used, highlighting the critical role of the SHA in the fabrication of SPPMs. By contrast, PCVImTf_2_N, which is also a poly(imidazolium) PIL but has a polar carboxamide group with H-bridge donor–acceptor function, could generate gradient porous structures as well. Thus, the SHAs in poly(1,2,4-triazolium) are the key sites, and PILs with strong H-bond donor–acceptor interactions are more viable for SPPM preparation by this method. Experimentally, the microphase separation process can be obviously observed by time-dependent laser scanning confocal microscopy images (Figure 5k). From theoretical simulation results, it is considered that microphase separation behavior might occur between the SHA (polar domain) and the hydrophobic counterion (apolar domain) upon water contact, which readily leads to porous structure in SPPMs. Conveniently, by designing the polarity of PIL cations or the hydrophobicity of PIL anions or both, the pore size of the SPPMs can be precisely controlled, which offers a water-based, simple, and efficient one-pot approach to fabricate SPPMs from a single PIL, in agreement with the concepts of “green processing”. At the same time, it has an inestimable guiding significance for the real-time preparation of SPPMs.

On the basis of this achievement, SPPMs were further functionalized with a single PIL component that bears chiral NH groups. It has been demonstrated that these chiral SPPMs are able to efficiently separate drug enantiomers (penicillamine) via directional H-bonding interactions.^50^ These aforementioned successful findings offer fascinating opportunities for engineering multifunctional SPPMs.

#### 3.4.2 Polycarbene-Bearing Nanoporous Membrane Actuators

In nature, the optimal performance of biological systems often depends on ingenious hierarchical structures. The development of artificial hierarchical structures capable of mimicking functions in biological systems requires a high order of structural complexity and multiple stimuli responsive functionality, yet the manufacture of such materials remains an enormous challenge.

**Figure 6:**
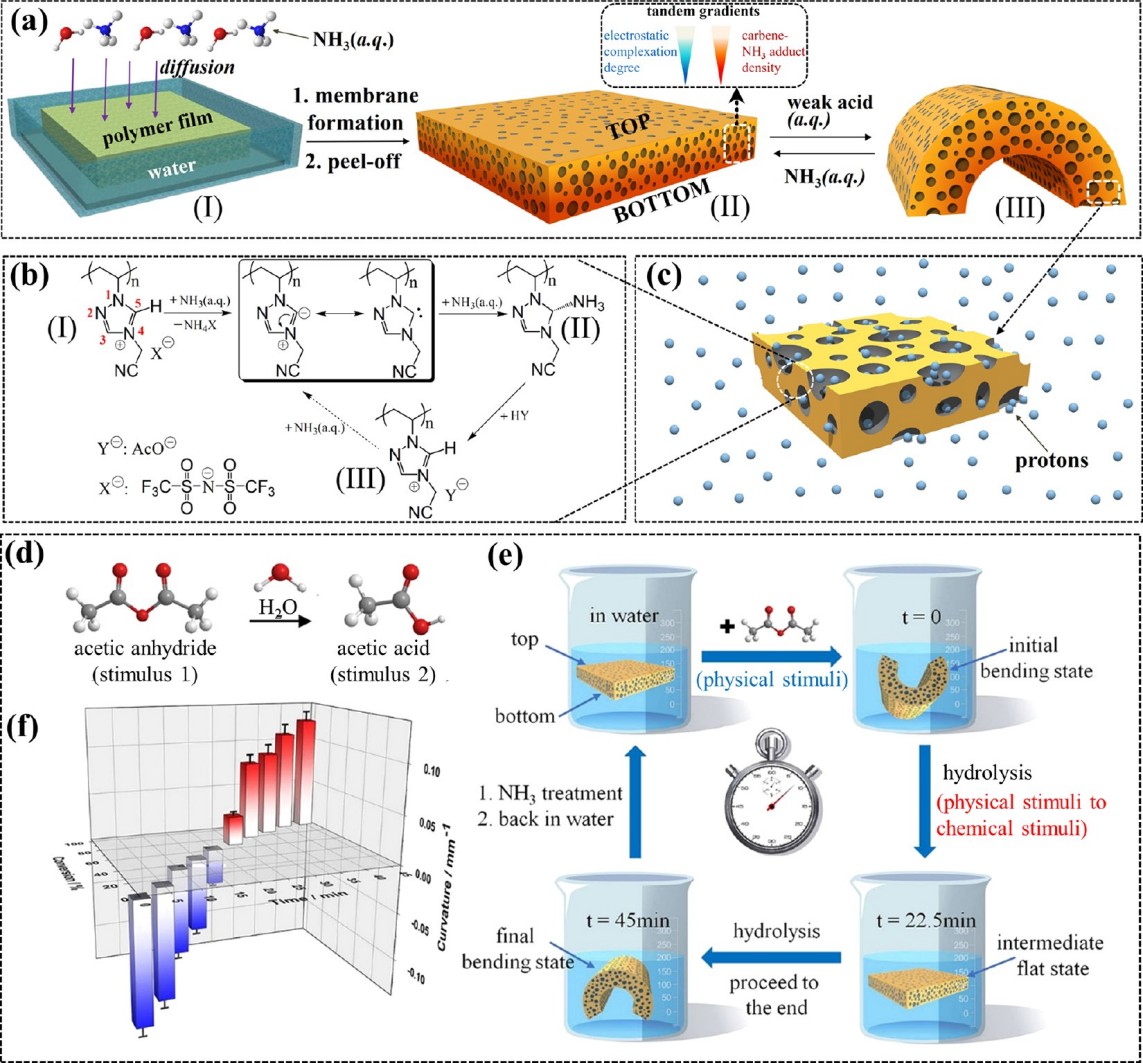
(a) Preparation and reversible conversion process of the membrane actuator. (b, c) Chemical structure transformation of the 1,2,4-triazolium PIL upon mild base and acid treatments. (d) Chemical equation of the hydrolysis of acetic anhydride. (e) Time-dependent shape change of the membrane actuator during the whole process of acetic anhydride hydrolysis. (f) 3D chart of membrane stripe curvature against the corresponding conversion process of acetic anhydride. Reproduced with permission from ref (2). Copyright 2018 Nature Communications.

In 2018, we established a synthetic route to a tandem-gradient nanoporous electrostatically cross-linked composite membrane using SHA chemistry of poly(1,2,4-triazolium). It has two structural gradients along the membrane cross-section, that is, the electrostatic complexation (EC) degree gradient and the carbene–NH_3_ adduct (CNA) density gradient.^2^ Ammonia molecules diffuse into the PIL–poly(acrylic acid) blend film from top to bottom and trigger a diffusion-induced EC gradient (Figure 6a).^51,52^ Meanwhile, the SHAs (C5-proton) of the 1,2,4-triazolium cations are active to undergo deprotonation to form NHCs. When NH_3_ is added in excess, it reacts with the active NHC *in situ* to form a CNA. From the viewpoint of mass transfer, an NH_3_ content gradient exists from top to bottom during the NH_3_ diffusion process. Hence, a density gradient of CNA units along the membrane cross-section was formed simultaneous to the EC gradient.

Upon treatment with a weak acid (e.g., acetic acid), the CNA can be reverted to the 1,2,4-triazolium cation. The chemistry of reversible formation–decomposition of CNA guides the membrane actuator for ultrasensitive proton detection in aqueous solution at the ppm level, which exceeds the performance of current soft proton actuators. As a control, the imidazolium-based PIL membrane bent only slightly at the same acetic acid concentration, while the pyridinium PIL membrane did not actuate at all due to the lack of SHAs on the pyridinium cation. This emphasizes the distinctive role of SHAs in the 1,2,4-triazolium cation.

Beyond recognition of protons, the competing actuation of the tandem-gradient architecture provides unexplored applications, such as visual readout of chemical reaction progress (Figure 6d–f). In the model process of acetic anhydride hydrolysis, the membrane strip was first flat in water and, after addition of acetic anhydride that swelled the membrane, bent with its top surface inward, which denotes the start of the reaction; the acetic anhydride molecules were stepwise consumed and transformed into acetic acid as product. Thus, the membrane strip was less swollen by acetic anhydride, while the interaction between the protons in solution and the CNA units in the membrane increased, which triggered the membrane to bend oppositely. A mathematical relation of time-dependent acetic anhydride conversion and membrane curvature was established (Figure 6f). Assisted by this chart, researchers can easily “read out” the invisible reaction process via the shape of the membrane strip. In brief, the smart membrane might be very useful for accurately monitoring the whole process of proton-involved reactions that include multiple steps and stimuli with competing responses. Moreover, based on the strong binding ability of the polycarbenes and their gradient distribution across the poly(1,2,4-triazolium) membrane, other gradients (e.g., metal clusters) of functional sites could be integrated into a membrane that already possesses an EC degree gradient, resulting in a single porous membrane carrying multiple gradients. The joint effect of these concurrent gradients in the membrane is supposed to enable high-level bionic actuation through continuously adapting to the dynamic multiple stimuli along an entire event.

## 4 Conclusion and Outlook

This Account has provided an overview of the chemical features, physicochemical properties, and exemplary applications of SHAs in 1,2,4-triazolium poly(ionic liquid)s as a molecular functionality platform that fosters materials chemistry innovation. The simple replacement of one carbon atom in the imidazolium cations by an electron-withdrawing nitrogen atom results in 1,2,4-triazolium cations with highly polarized C5–H bonds, which come with a drastically new chemistry. As a key feature, the SHAs of the C–H bond display different properties as hydrogen bond donors, Bro̷nsted acids, and polycarbene precursors. Such a unique profile of SHAs and the intrinsic features of heterocyclic PILs provide a powerful tool to expand materials chemistry.

Some illustrative model applications related to SHAs are highlighted here. The hydrogen bond structure and strength between SHAs and water molecules affect the self-assembly behavior of PIL chains in an aqueous phase, which enables the subtle manipulation of the interior and outer morphology and surface properties of self-organized polymer nanocolloids in aqueous phase. Thanks to the cooperativity of the metal cluster stabilization effects stemming from polycarbene formation, N-coordination, and ionic interactions, metal clusters with their size controllable down to sub-nanometer scale were synthesized. In a further application case, the *in situ* released free protons from the SHA act as an organocatalyst to lower the molecular formation energy at room temperature. By dynamic combinatorial chemistry, a variety of imine-linked materials is formed, while the wanted crystalline porous system is supported by lowering its crystallization energy barrier by PIL surface stabilization. Meanwhile, the “salting-out” effect of the PILs caused accelerated precipitation but still unperturbed crystallization of solutes. In a last set of cases, the versatile supramolecular interactions of SHAs and their synergistic effect enabled the construction and functionalization of smart supramolecular membranes with switchable pore opening and closing and multigradient structures. These membranes have exhibited impressive functions ranging from responsive optical properties to chiral separation, proton recognition, and real-time chemical reaction monitoring and actuation.

Although the concept of the SHA is yet to be fully developed, the intriguing physicochemical properties and the successful examples of applications exemplified in this Account portend strong prospects for SHAs as a platform tool for self-organization and related materials functionality, very similar to the “supramolecular polymers”.^53^ We indeed believe that the combination of the SHA donor–acceptor structure, strong ionic interactions, and possible other interactions via structured water or polycarbene recognition adds up to a secondary interaction tool; such a tool can drive solution entropy effects and the coupled self-organization that is otherwise mostly known in water as the magic solvent. One can foresee that by rational design, a myriad of PIL-based functional materials at various size scales may find real-life use, such as in (chiral) separation, smart devices, and catalysis.

The fundamental importance of SHAs in PIL systems could only be touched upon in this Account, and challenges remain in the quest to explore the kinetic roles of SHAs in chemical processes, that is, the formation and diffusion of active species, interactions, and reaction mechanisms during the processes of chemical transformations and material fabrication. From this point of view, modeling and simulations for identifying the contribution of SHA-related interactions and lab reactions are parallel approaches. In addition, manipulating the activity of the SHA through engineering the chemical microenvironment around the SHA (e.g., the chemical structure of the heterocyclic cations, substituent groups, and counteranions) and the underlying mechanism is worth in-depth study. We hope that the rather cohesive body of first experiments will inspire ideas and drive new applications to harness SHA chemistry within a wider research scope.

## Supporting Information Available

Chemical structures and abbreviations of ionic liquids and poly(ionic liquid)s mentioned in this Account (PDF)
