# Supplementary material for: Smart Hydrogen Atoms in Heterocyclic Cations of 1,2,4-Triazolium-Type Poly(ionic liquid)s
Source: Acc Chem Res. 2022 Dec 5;55(24):3675–87. doi: 10.1021/acs.accounts.2c00430 (PMC9774662; doi:10.1021/acs.accounts.2c00430)
Supplement: Supplementary file 1 — ar2c00430_si_001.pdf [file ar2c00430_si_001.pdf]

Supporting Information

Smart Hydrogen Atoms in Heterocyclic Cations of 1,2,4-Triazolium-Type Poly(ionic liquid)s

*Si-hua Liu,[a] Hong Wang,[*b] Jian-ke Sun,[*a] Markus Antonietti,[c] Jiayin Yuan[*d]*

[a] Si-hua Liu, Jian-ke Sun − *MOE Key Laboratory of Cluster Science, Beijing Key Laboratory of Photoelectronic/Electrophotonic Conversion Materials, School of Chemistry and Chemical Engineering, Beijing Institute of Technology, Beijing 102488, P. R. China*
[b] Hong Wang − *Key Laboratory of Functional Polymer Materials (Ministry of Education), Institute of Polymer Chemistry, College of Chemistry, Nankai University, Tianjin 300071, P. R. China*
[c] Markus Antonietti − *Department of Colloid Chemistry, Max-Planck Institute of Colloids and Interfaces, Research Campus Golm, Am Mühlenberg 1, 14476 Potsdam, Germany*.
[d] Jiayin Yuan − *Department of Materials and Environmental Chemistry, Stockholm University, Stockholm, 10691 Swede*

Corresponding author's email address
Hong Wang; Email: hongwang1104@nankai.edu.cn
Jian-ke Sun; Email: jiankesun@bit.edu.cn
Jiayin Yuan; Email: jiayin.yuan@mmk.su.se

Key References

- Sun, J.-K.; Kochovski, Z.; Zhang, W.-Y.; Kirmse, H.; Lu, Y.; Antonietti, M.; Yuan, J. General synthetic route toward highly dispersed metal clusters enabled by poly(ionic liquid)s. *J. Am. Chem. Soc.* **2017**, 139(26), 8971-8976.

- Sun, J.-K.; Zhang, W.; Guterman, R.; Lin, H.-J.; Yuan, J. Porous polycarbene-bearing membrane actuator for ultrasensitive weak-acid detection and real-time chemical reaction monitoring. *Nat. Commun.* **2018**, 9, 1717.

- Shao, Y.; Wang, Y. L.; Li, X.; Kheirabad, A. K.; Zhao, Q.; Yuan, J.; Wang, H. Crosslinking of a single poly(ionic liquid) by water into porous supramolecular membranes. *Angew. Chem. Int. Ed.* **2020**, 59, 17187-17191.

- Zhang, S.-Y.; Miao, H.; Zhang, H.-M.; Zhou, J.-H.; Zhuang, Q.; Zeng, Y.-J.; Gao, Z.; Yuan, J.; Sun, J.-K. Accelerating crystallization of open organic materials by poly(ionic liquid)s. *Angew. Chem. Int. Ed.* **2020**, 59, 22109-22116.

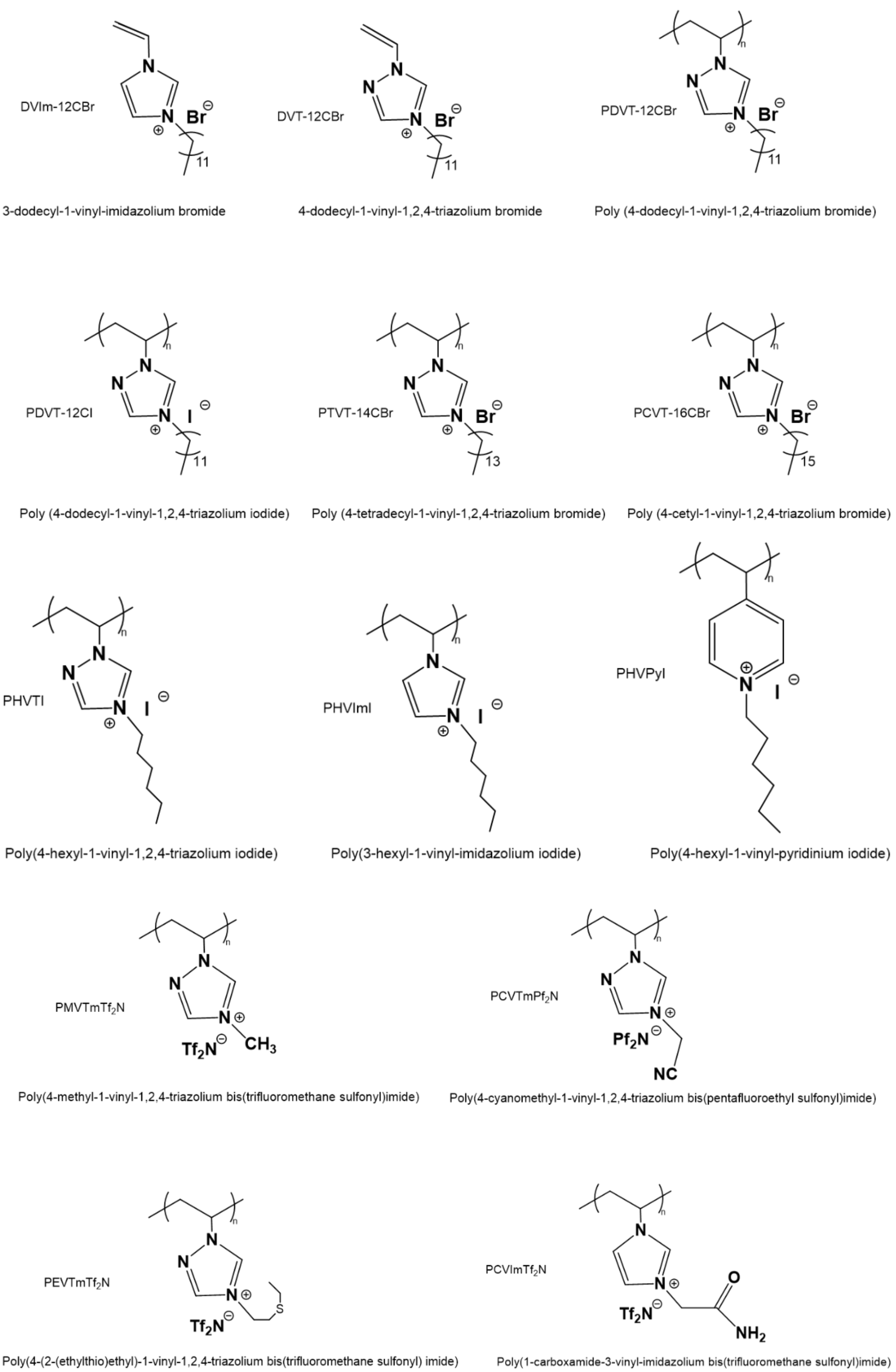

Figure S1. Chemical structural information and their abbreviations of ionic liquids and poly(ionic liquid)s mentioned in this Account.
